# Comparing the portrayal of #autism and #neurodiversity on TikTok: creators, content, and representation

**DOI:** 10.1007/s10354-025-01076-w

**Published:** 2025-03-11

**Authors:** Verena Steiner-Hofbauer, Yvette Annabel Pintér, Gloria Mittmann

**Affiliations:** Research Centre Transitional Psychiatry, Karl Landsteiner University of Health Sciences, Dr. Karl-Dorrek-Straße 30, 3500 Krems, Austria

**Keywords:** Social media, ASD, Mental illness, Stigma, Depiction

## Abstract

**Background:**

Social media is a significant source of information on health-related topics and neurodevelopmental disorders such as autism spectrum disorder (ASD). The public perception of ASD, as reflected on social media, can raise awareness but also increase stigma. This study examined ASD portrayal on TikTok, focusing on neurodiversity, content themes, creator identities, and the depiction of autistic individuals.

**Materials and methods:**

This exploratory study analyzed 100 TikTok videos: the 50 most-watched for #autism and the 50 most-watched for both #autism and #neurodiversity. The study reviewed metadata and content using publicly available data.

**Results:**

Videos from the #autism sample encompassed 97% of all views and primarily portrayed entertaining content. Neurodiversity videos were more educational and less popular. Creators and portrayed individuals were primarily white. Adult autistic individuals are more ferequently represented in the #neurodiversity sample (30%), but children sill appear frequently (30% in the # neurodiversity and 38% in the #autism sample). Healthcare professionals (HCPs) were absent in the autism sample but appeared in 32% of neurodiversity videos.

**Conclusion:**

The portrayal of ASD differed widely in both samples. Both samples underrepresented ethnic minorities. As TikTok shapes public perception of ASD, HCPs should be aware of trending ASD-related content on TikTok in order to be able to combat misinformation.

## Introduction

According to the ICD-11 (International Statistical Classification of Diseases and Related Health Problems, 11th edition), autism spectrum disorder (ASD) is a neurodevelopmental condition characterized by early-onset difficulties in initiating and sustaining reciprocal social interaction and communication as well as restricted, repetitive, and inflexible patterns of behavior that surpass age-related expectations [32]. ASD is considered a spectrum due to the substantial variability in symptom severity and manifestation among individuals [14]. Recent definitions in the DSM‑5 and ICD-11 frame ASD as a continuous spectrum without specific subtypes, instead measuring core symptoms dimensionally from mild to severe [24].

Over time, the understanding of ASD has evolved, giving rise to movements such as the neurodiversity movement, which advocates a more inclusive perspective of autism as one among many naturally occurring variations in human cognition [5, 15]. From this standpoint, neurodivergent attributes are viewed as structural and functional brain variations that become advantageous or disadvantageous depending on the environmental context. In “environments structured in accordance with neurotypical perspectives” [27, p. 560], these variations may be pathologized, but in environments that accommodate such differences, the same traits do not necessarily constitute impairments [27]. When neurotypical perspectives overshadow the strengths of neurodiverse individuals, it can foster shame, low self-worth, and related mental health challenges [27, 30]. The neurodiversity movement thus seeks to foster supportive ecological niches, promoting acceptance and pride rather than “treating” or “curing” neurodivergence [6]. The term “neurodiversity,” coined by Judy Singer, is often applied in contexts of ASD and other neurodevelopmental conditions such as ADHD or learning disabilities [31].

The “conventional medical paradigm” often employs a deficit-based view of autism, focusing on impairments and the need for intervention. This paradigm is a key element for research, diagnosis, and (development of) clinical practice that has been evidently helpful for many. Both viewpoints, even if they seem different, agree that autism is biological in nature and both bear a common conception that support, clinical treatments, and research are important to improve the lives of neurodiverse people [25, 27].

Social media has emerged as a key source of health-related information, including content on neurodevelopmental disorders like ASD [34]. The portrayals of these conditions online both influence and reflect public perceptions [28]. While accurate and balanced representations can enhance understanding, inaccurate or exaggerated depictions may perpetuate stigma and stereotypes [3, 28]. Mainstream media’s portrayal of autism can provide educational value but often has a narrow focus, challenging stereotypes, and highlights only certain aspects of autistic experiences [12]. For instance, many television series and films depict autistic characters as white males with savant syndrome, contributing to a limited public image of autism [21]. Such narrow portrayals may stem from the underrepresentation of autistic individuals and experts in content creation. In contrast, research suggests that social media offers a more supportive environment, possibly due to increased self-representation by autistic individuals [20]. Their inclusion broadens the range of presented characteristics, creating more accurate and positive representations.

TikTok, launched globally in 2017, now reaches over 1.677 billion users worldwide, including 150 million in the United States. Its rising popularity as a platform for ASD-related content offers new opportunities for representation but also heightens the risk of disseminating misleading or non-evidence-based information. Although a few studies have examined ASD-related content on TikTok, existing research has focused on highly viewed videos to understand what users encounter online. Additionally, TikTok serves as a platform for autistic individuals to share personal experiences, build supportive communities, and shape outsiders’ perceptions [2, 8].

A recent Norwegian study found that many ASD-related TikTok videos contain misleading or non-evidence-based information, with no input from healthcare professionals and few videos offering genuinely useful guidance [10]. Another study confirmed these findings and further noted the lack of representation of autistic individuals from black, indigenous, and other minority communities [8], reflecting broader underrepresentation across social media platforms. Because much ASD-related content on TikTok is rooted in personal experience, it is essential to investigate who creates this content, how they portray autism, and how they self-identify. Many content creators align with the neurodiversity perspective, embracing neurodivergent identities and contributing to destigmatization and advocacy [19]. Given the influence of the neurodiversity movement, understanding its representation on widely used platforms like TikTok is critical.

Previous research on ASD-related TikTok content has primarily addressed the reach and accuracy of informational videos, finding a prevalence of inaccuracies and overgeneralizations [2, 10]. To our knowledge, no study has specifically examined how autism is represented on TikTok through the lens of neurodiversity. Thus, the aim of this article is to analyze ASD-related video content on TikTok, focusing on the portrayal of neurodiversity in relation to autism.

## Methods

This exploratory study aimed to evaluate ASD-related content on the social media platform TikTok with the primary focus on the portrayal of ASD and its link to neurodiversity. The methods of this study are primarily based on the cross-sectional study of Anthony Yeung, Enoch Ng, and Elia Abi-Jaoude, which examined the quality of ADHD-related content on TikTok, and on the content analysis of ASD-related YouTube Videos by Schwab Bakombo, Paulette Ewalefo, and Anne T. M. Konkle [3, 33].

### Approach

For data collection, a new account was created on TikTok to avoid bias from TikTok’s recommendation algorithm which is based on the user’s previous viewing history [34]. The data collection was conducted on the 25 July 2023 and was executed by searching once for the hashtag #autism and once for both hashtags #autism and #neurodiversity in the TikTok application. The raw data set included the first 100 videos which appeared under the hashtag #autism as well as the first 100 videos appearing under the hashtags #autism and #neurodiversity.

A video’s popularity can be measured by the number of views and likes it receives. When searching for a hashtag, the videos with the highest number of views and likes appear first, followed by less-viewed and less-liked videos [17].

The inclusion criteria were that the videos had to be ASD related, had to be in English language, and had to contain either #autism or both #autism and #neurodiversity. Regarding exclusion criteria, videos that were not ASD related, not in English language, or were duplicates were not included in the final dataset for analysis.

**Fig. 1:**
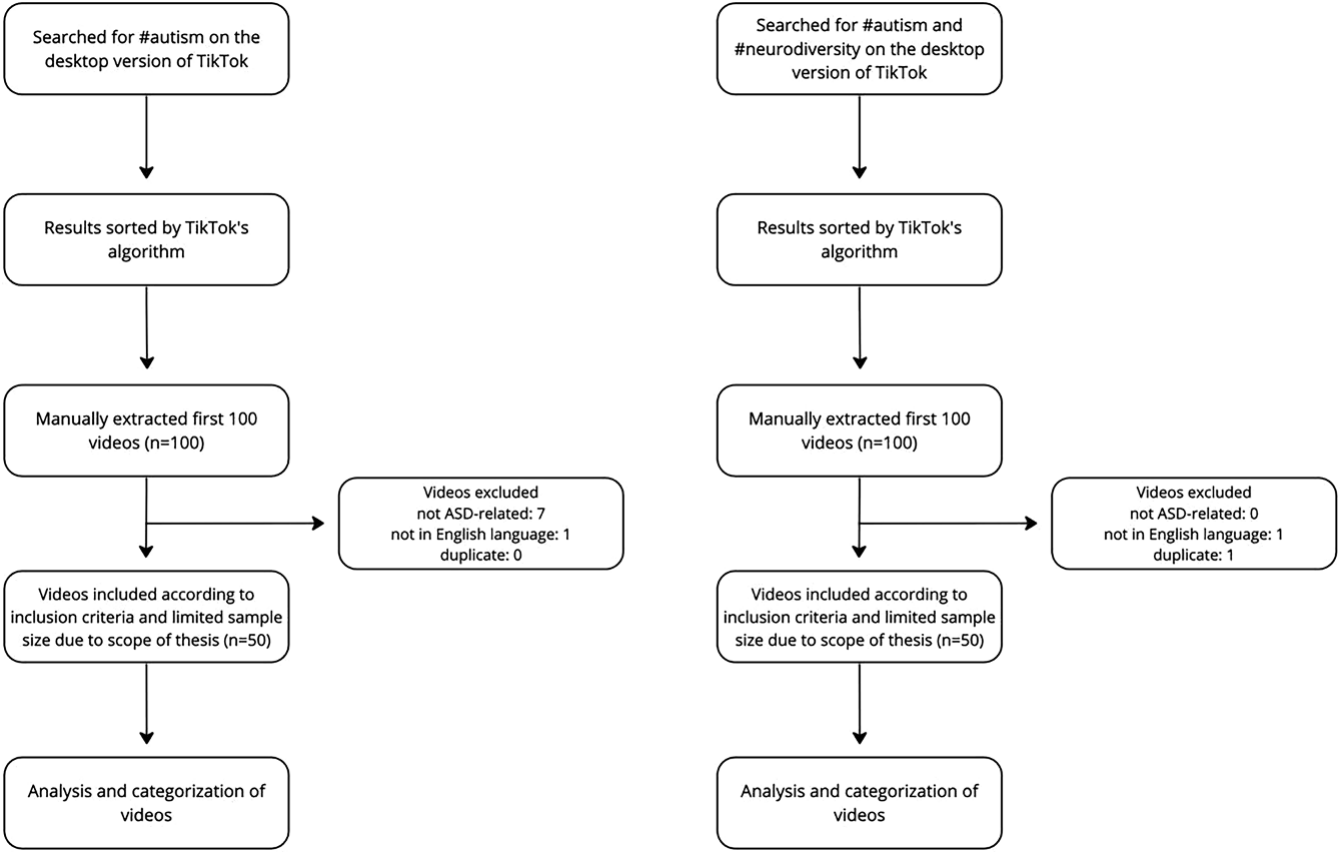
Flowchart of the sampling process

Figure 1 shows the method used to create the final dataset after applying the inclusion and exclusion criteria.

### Selection process

The first 100 videos were manually saved in an Excel (Redmond, WA: Microsoft, 2021) sheet, where the first videos which met the inclusion criteria were analyzed until the aspired sample size of 50 was reached. If a video did not meet the inclusion criteria it was removed, and the next video from the results list was selected. A total of eight videos were removed in the process of obtaining the final dataset for the first sample containing #autism. Seven out of eight videos were removed as they were not ASD related, meaning that the #autism hashtag had been added to the video even though the video was not related to autism. One video was excluded as it was not in English language. Regarding the selection process for the second sample with both #autism and #neurodiversity, two videos did not meet the eligibility criteria as one was not in English language and the other was a duplicate.

### Process of sample analysis

The videos of the final dataset were analyzed for their metadata, their creators, and content. For the metadata, the number of views, likes, shares, and comments were collected to determine their public reach on the platform. For the content analysis, the videos were allocated to the following categories: *education, people and blogs, film and animation, entertainment*, and *news*. The themes were adopted from a recent study which analyzed ASD-related videos on YouTube [3]. Moreover, the differences in content were analyzed between the two sample sets. Therein, the focus was to determine how many of the educational videos were scientifically correct and whether they were created by healthcare professionals (HCPs) or not. Additionally, the most frequently portrayed symptoms of ASD were noted and analyzed. Furthermore, the creator identities were analyzed. This was done by noting information which the creators publicly displayed in their biographies visible on their profiles and by analyzing their appearance and information given in the video. The two main focuses regarding the creator identities were to distinguish whether they were HCPs or non-HCPs and whether the creator was autistic or not. Additional information such as the creator’s ethnicity, approximate age, gender, and their relation to the person portrayed in the video were noted. As for the autistic person portrayed in the video, their ethnicity, approximate age, and gender was analyzed.

Lastly, the hashtags section of each video was analyzed. The primary focus was to identify how many of the videos appearing under the #autism hashtag contained additional hashtags related to neurodiversity to analyze which further hashtags the creator used for their video. The same approach was used for the videos appearing under both #autism and #neurodiversity. This analysis should give new insights into how the creators wish to portray their ASD-related video and whether they represent the neurodiversity movement.

## Results

### Baseline video characteristics

The 100 videos making up the final dataset had a total of 764.7 million views. The first selected top 50 videos featuring #autism had 738.1 million views. The most-watched video had 60.9 million views, while the video which was watched the least had 2.6 million views. In comparison, the other 50 videos featuring the additional #neurodiversity hashtag had a total of 26.6 million views. The most popular video reached 16.1 million views, while the least watched video had 2125 views. All videos in the final dataset received a total of 123.2 million likes. The videos with only the #autism hashtag received 120.3 million likes. The videos which additionally featured the #neurodiversity hashtag received a notably smaller number of likes at 2.9 million.

### Videos characterized by content

**Fig. 2:**
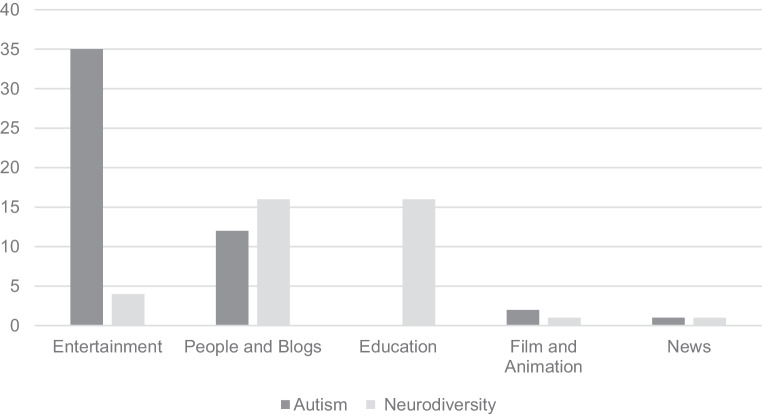
Number of videos in five different content theme categories, #autsim (autism) *n* = 50, #autsim and #neurodiversity (neurodiversity) *n* = 50

The content analysis showed that discussions related to ASD primarily revolved around three themes: “people and blogs,” “entertainment,” and “education.” There was a difference regarding the most popular theme between the two sampling groups, with #autism mainly featuring “entertainment” and #neurodiversity mainly featuring “education” and “people and blogs” (Fig. 2).

### Characterization of predominantly featured symptoms

In the autism sample, the most frequently featured ICD-11 symptoms of ASD were deficits in the ability to initiate and sustain reciprocal social interaction and communication (18%), followed by repetitive and stereotyped motor movements such as an atypical gait (12%) as well as excessive hyper- or hyposensitivity to sensory stimuli or an unusual interest in sensory stimulus (12%). In the neurodiversity sample, a notable number of videos highlighted the latter and displayed this symptom mostly in terms of an educational content (26%). On the contrary, the autism sample portrayed the symptom within an entertaining or personal experiential framework. Deficits in initiating and sustaining reciprocal social interaction and communication were the second most highlighted symptoms in the neurodiversity sample (12%). Less frequently highlighted symptoms were persistent preoccupation with one or more special interests (4%) and excessive and ritualized patterns of behavior such as lining up specific objects in a particular way (2%).

### Videos characterized by creator identities

Table 1 shows the data of video creators of videos featuring #autism (*n* = 50) and the 50 most popular videos featuring both #autism and #neurodiversity.

**Table 1:** Percentage of videos featuring creators with respect to age, gender, race, autistic/not autistic, healthcare professional (HCP)/non-HCP. *n* = 50 #autism, *n* = 50 #autism and #neurodiversity

	Autism sample (%)	Neurodiversity sample (%)
*Age category of creator*		
Child	0	2
Young adult	36	20
Adult	54	70
Not known	10	8
*Gender of creator*		
Male	20	26
Female	70	64
Diverse	2	2
Not known	8	8
*Race of creator*		
White	72	86
Person of color	10	6
Not known	18	8
*Creator autistic yes/no/not known*		
Yes	40	58
No	58	38
Not known	2	4
*Creator HCP yes/no/not known*		
Yes	0	32
No	100	68
Not known	0	0

### Creator identities in relation to the portrayed autistic person

In the autism sample, 40% of the creator identities were autistic and publicly displayed their diagnosis, either in a short description on their profiles or by mentioning it in one or several of their videos; 30 videos (60%) had creators who were not autistic and who had different relations to the portrayed autistic person. In 40% of the cases, the portrayed autistic person was the child of the content creator. In 37%, the content creator portrayed their autistic brother or sister, while in 17%, another relation was present, for example, a public person or movie character. In 2% of cases, the relation was not known. In the neurodiversity sample, 38% of the videos were published by content creators who were not autistic and in 52%, the portrayed autistic person was their child. In 38%, the content creator had other relations to the portrayed autistic person or was talking about ASD in general, without depiction of an autistic individual in their videos. In 10% of the videos, the relation to the creator was not known.

### Characteristics of the portrayed autistic person

Table 2 represents the characteristics of the autistic person portrayed in the videos of the autism sample (*n* = 50 #autism) and the neurodiversity sample (*n* = 50 #autism and #neurodiversity).

**Table 2:** Percentage of videos featuring autistic individuals with respect to age, gender, race, and healthcare professional (HCP)/non-HCP. *n* = 50 #autism, *n* = 50 #autism and #neurodiversity

	Autism sample (%)	Neurodiversity sample (%)
*Age category of autistic individual*		
Child	38	30
Young adult	36	20
Adult	20	30
Not known	6	20
*Gender of autistic individual*		
Male	42	46
Female	48	28
Diverse	2	2
Not known	8	24
*Race of autistic individual*		
White	70	76
Person of color	20	6
Not known	10	18
*Autistic individual HCP/non-HCP*		
Yes	0	18
No	100	82

### Analysis of the hashtag section

In the autism sample, 40% of the hashtags related to TikTok’s “For You” page such as #fyp, #fup, or #foryou. In 36% of the 50 videos, hashtags related to autism awareness and acceptance such as #autismawareness and #autismacceptance. In 14% of the videos from the autism sample, trending hashtags relating to comedy or humor such as #funny, #meme, or #comedy were used. In 8% of the analyzed videos, hashtags related to neurodivergence (but not #neurodiversity) and one video used hashtags relating to disability. In the neurodiversity sample, the most highly trending hashtags were related to autism awareness and acceptance (54%). Only 12% of the videos from the neurodiversity sample used hashtags related to TikTok’s “For You” page, and none of the videos used hashtags related to comedy or humor. In 6% of the videos from the neurodiversity sample, hashtags related to disability.

## Discussion

There is a growing body of literature examining how ASD is depicted in various forms of media, including film, television, and literature [12, 20]. More recently, attention has shifted to social media platforms such as YouTube and TikTok, with numerous studies evaluating the reach, accuracy, and educational quality of ASD-related content [2, 3]. In this context, the present study focused on content themes, creator identities, the portrayal of autistic individuals, and the representation of neurodiversity on TikTok.

Our findings revealed notable differences between videos retrieved using only #autism (the autism sample) and those retrieved using both #autism and #neurodiversity (the neurodiversity sample). While content in the autism sample largely fell under the “entertainment” theme, the neurodiversity sample produced more educational material. The inclusion of #neurodiversity significantly influenced the content profile, shifting from humor and comedy toward more informative videos. This distinction aligns with prior work on other platforms; for example, a 2019 YouTube study identified “education” as the dominant theme among ASD-related videos [3]. These results suggest that platform- and hashtag-specific searches can yield markedly different portrayals of ASD.

In terms of viewer reach, the autism sample achieved a significantly larger audience. Given that many individuals rely on TikTok for health-related information [13], this discrepancy is concerning. Educational content is less visible, meaning that users searching for autism-related information are more likely to encounter humor-based videos rather than accurate, informative material. Such patterns may perpetuate misconceptions, as these videos often highlight only a few ASD symptoms without contextualization. Specifically, the autism sample frequently depicted deficits in social interaction and communication, while the neurodiversity sample emphasized sensory sensitivities. Although the neurodiversity sample presented symptoms in an educational framework—often featuring autistic individuals or healthcare professionals (HCPs)—both samples offered a limited symptom range. Consequently, viewers may erroneously conclude that experiencing one or two highlighted symptoms is sufficient for an ASD diagnosis, ignoring the broader diagnostic criteria outlined in ICD-11 [32].

This selective focus on particular symptoms mirrors issues noted for other health conditions on social media, where creators highlight more dramatic symptoms to attract viewers, potentially contributing to the spread of misinformation [7, 23]. Such practices underscore the importance of promoting accurate, evidence-based content on social media to counteract sensationalized and misleading portrayals.

Examining creator identities, female adults predominated in both samples, consistent with research on other mental health-related topics on TikTok [34]. However, a key difference emerged regarding HCP involvement: the autism sample included no HCP creators, reflecting previous findings of minimal professional engagement online [10, 33]. In contrast, 30% of the neurodiversity sample’s videos featured HCPs, underscoring the potential value of professional involvement for improving public awareness and reducing misinformation about ASD [2, 11].

Differences in autistic creator representation also highlight the importance of authentic self-representation. The neurodiversity sample included more autistic creators, contributing to a more diverse and positive portrayal of ASD [20]. While the autism sample included slightly more female autistic individuals, the neurodiversity sample showed a higher proportion of male autistic individuals, aligning with known prevalence ratios [32]. Both samples lacked adequate representation of ethnic minorities, reflecting broader inequalities in diagnosis and access to autism services [4, 16, 26]. Increased representation of minority groups on social media is critical to foster inclusion and improve mutual understanding within the autistic community [8].

Children were often depicted in our sample of TikTok videos. In the autism sample, children were the most frequently portrayed age group (children: 38%, young adults: 36%, adults: 20%). This is consistent with previous findings that media often infantilize autism and underrepresent autistic adults [1, 29], which is problematic given that many autistic adults remain undiagnosed or self-diagnose, with significant emotional consequences [9, 22]. Overall, it is important to highlight that the publication of images and videos of underage children on social media is a distinct issue with numerous legal and ethical pitfalls that go beyond the scope of this work.

Additional differences emerged regarding reach and popularity. The autism sample accounted for 97% of total video views, suggesting that entertaining, humor-focused content receives more exposure than educational material. This preference may be driven by the strategic use of hashtags like #fyp, which boosts views but lacks any ASD-specific educational value [18]. By comparison, the neurodiversity sample prioritized hashtags related to autism awareness and acceptance over those aimed solely at increasing visibility. Research has emphasized the need for a diverse, personalized, and non-stigmatizing representation of mental illnesses in the media. But it is important to note that social media, primarily designed for self-expression and connecting with like-minded individuals, provides a platform for exchange, interaction, and a sense of belonging [20]. Therefore, it is important to raise awareness in the general population that social media such as TikTok might not be the best place to seek health-related information.

Although the concept of neurodiversity appeared in both samples, it was less prevalent in the autism sample. Many videos of the autism sample depicted autism humorously, potentially stigmatizing autistic individuals and infringing upon their dignity, privacy, and personal integrity. Despite including hashtags like #autismacceptance and #autismawareness, the intention of these videos seemed geared toward entertainment and gaining likes. In contrast, the neurodiversity sample included more autistic creators and HCPs, suggesting that when neurodiverse voices guide content, portrayals may be more accurate, respectful, and supportive.

## Limitations

This study focused solely on TikTok and did not examine other social media platforms, which may limit the generalizability of the findings. Furthermore, TikTok’s rapidly evolving content landscape means that top-viewed videos can change quickly. Although we analyzed 100 videos, representing the most-viewed content at the time, this sample may still be considered small given the platform’s vast output. Additionally, no neurodivergent coresearchers were involved in the analysis, though we employed objective criteria to reduce bias.

## Conclusion

This study provides insights into the current portrayal of ASD on TikTok. The findings underscore the need for strategies to elevate educational, evidence-based content and to encourage the involvement of HCPs and autistic individuals in content creation. Equitable and ethical representation—particularly concerning children and ethnic minorities—is essential. Addressing these issues may help social media to evolve into a more inclusive, accurate, and supportive space for the neurodiverse community. The study reveals that TikTok’s portrayal of autism spectrum disorder (ASD) is predominantly entertainment focused, lacking educational value. Depicting misleading or exaggerated symptoms or humorous scenes without a clear connection to autism could potentially perpetuate misconceptions and stigma. Such content can undermine the dignity and rights of autistic individuals, conflicting with the principles of the Convention on the Rights of Persons with Disabilities (CRPD), which emphasize respect, non-discrimination, and full societal participation.
